# Should Now Receive Consideration

**Published:** 1897-09

**Authors:** 


### SHOULD NOW RECEIVE CONSIDERATION.

The recent experiences noted at Kirk Park, New York, and at Carnegie, Pa., trotting-tracks, where horses were directed by the judges to finish the race in the face of protests of the owners of said animals, who wished to withdraw them because of their unfit physical condition for such trials, and where in both instances death of very valuable animals followed, awakens a new thought and suggests a new field of employment for veterinarians. In all such cases an unbiased veterinarian, not connected with the track or interested in the races in any manner, should have been summoned and his decision in this matter been final. A careful physical examination of these animals would, no doubt, have determined their condition, and their lives have been prolonged had they not been compelled to finish the races. It would be well for all true lovers of horse-racing to consider the urgent necessity of providing for just such cases as these in the prompt selection of reputable and equipped veterinarians to decide matters, and thus render justice to all concerned, without imperilling valuable property unnecessarily.

